# Characterization of the Two Intra-Individual Sequence Variants in the 18S rRNA Gene in the Plant Parasitic Nematode, *Rotylenchulus reniformis*

**DOI:** 10.1371/journal.pone.0060891

**Published:** 2013-04-11

**Authors:** Seloame T. Nyaku, Venkateswara R. Sripathi, Ramesh V. Kantety, Yong Q. Gu, Kathy Lawrence, Govind C. Sharma

**Affiliations:** Department of Biological and Environmental Sciences, Alabama A&M University, Normal, Alabama, United States of America; Genomics and Gene Discovery Research Unit, USDA-ARS, Albany, California, United States of America; Department of Entomology and Plant Pathology, Auburn University, Auburn, Alabama, United States of America; Virginia Commonwealth University, United States of America

## Abstract

The *18S* rRNA gene is fundamental to cellular and organismal protein synthesis and because of its stable persistence through generations it is also used in phylogenetic analysis among taxa. Sequence variation in this gene within a single species is rare, but it has been observed in few metazoan organisms. More frequently it has mostly been reported in the non-transcribed spacer region. Here, we have identified two sequence variants within the near full coding region of *18S* rRNA gene from a single reniform nematode (RN) *Rotylenchulus reniformis* labeled as reniform nematode variant 1 (RN_VAR1) and variant 2 (RN_VAR2). All sequences from three of the four isolates had both RN variants in their sequences; however, isolate 13B had only RN variant 2 sequence. Specific variable base sites (96 or 5.5%) were found within the *18S* rRNA gene that can clearly distinguish the two *18S* rDNA variants of RN, in 11 (25.0%) and 33 (75.0%) of the 44 RN clones, for RN_VAR1 and RN_VAR2, respectively. Neighbor-joining trees show that the RN_VAR1 is very similar to the previously existing *R. reniformis* sequence in GenBank, while the RN_VAR2 sequence is more divergent. This is the first report of the identification of two major variants of the *18S* rRNA gene in the same single RN, and documents the specific base variation between the two variants, and hypothesizes on simultaneous co-existence of these two variants for this gene.

## Introduction

The ribosomal DNA (rDNA) plays a pivotal role in protein synthesis in eukaryotes and changes in these genes can profoundly affect ecological interactions, host range, trophic production, and thus the overall growth and resource requirement of the organism [1]. Therefore, rRNA variation has effects far beyond cell biology alone. The rDNA region is composed of numerous copies of tandemly repeated transcription units within the genome. It consists of the *18S*, *5.8S*, and *28S* genes as well as internal and external transcribed spacers (ITS and ETS) [2]. Concerted evolution is implicated in the conservative nature of the repeats [3], [4], through molecular mechanisms such as gene conversion [4], [5] and unequal crossing-over [6]. Since various regions of this gene evolve at different rates [2], the sequence homology provides comparative phylogenetic differences between or within organisms. Intra and inter variations within the *18S* rRNA gene sequences of species have been observed in genomes of some organisms [7], [8], [9] often attributed to frequent recombination events involving unequal crossovers and gene conversions producing rDNA units having similar sequences [9].

The RN is endemic to the southern U.S. and in a number of tropical and subtropical regions of the world [10], including Africa, Asia and Australia [11]. The absence or infrequent usage of non-hosts, such as corn (*Zea mays*) in crop rotations with cotton (*Gossypium hirsutum*), aggravates the incidence of RN. This organism also has a diverse host range that includes nearly 300 plant species [10], [12] and possesses the ability to live in prolonged dry and harsh environments [12]. This anhydrobiosis feature facilitates its dispersal to distant areas by dust storms [13]. Considerable damage is caused by the RN and this reduces cotton production in Alabama [14], Louisiana [15], and Mississippi [16] where in 2006 approximately 328,073 bales of cotton or $128 million in revenue were lost. Monoculture is the main reason why RN populations keep increasing [17], [18].

The rDNA serves as a key molecular marker for establishing phylogenetic relationships. Within a number of organisms, the rRNA genes can provide the evolutionary history [19]. This gene has variable regions which have been used to elucidate molecular phylogenies in organisms including those of free-living, animal, and plant parasitic nematodes [20]–[22]. In choosing a molecular marker or region for phylogenetic analysis, considerable care is required to accurately reflect the true evolutionary relationships among taxa. The *18S* rDNA region, because of its conservative nature, has been a staple in such phylogenic analyses within and between various taxa [23]. The presence of more than one variant therefore, is an infrequent rarity and demands further investigation. The objectives of this study were to: 1. Elucidate the full-length *18S* rRNA gene nucleotide sequence of a single RN, and 2. Characterize the 18S rRNA gene for occurrence of any potential variation in this gene.

## Results

**Figure 1:**
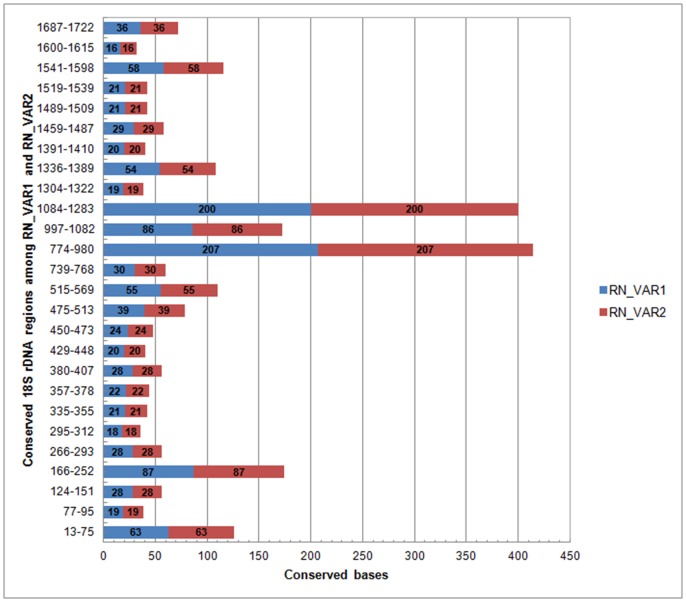
Twenty-six conserved regions within the 18S rRNA gene of four juvenile female RNs.

Amplification of RN DNA using primer pairs SSUF05 and SSUR81 gave a band of approximately 1800 bp and 49 clones were sequenced from four female RNs. Sequencing of the 1800 bp band was accomplished by using a combination of six primers (**Table 1**). Multiple sequence alignment (MSA) was performed using ClustalW (http://align.genome.jp/ ) with all the clone sequences generated from each nematode using default parameters and viewed using Bioedit software [24]. The MSA table is provided as an additional file (**Table S1**). Two types of *18S* rDNA sequences were observed from MSA, these were labeled as RN_VAR1 and RN_VAR2 referred to as the first and second variants of the *18S* rDNA sequence of the RN, respectively. Five of the clones (SSU1B2, SSU1B3, SSU1B7, SSU1B9, and SSU1B10) were identified as putative chimeras because these had their preference scores above 1.0. These clones were therefore not used for further analysis. The 44 clone sequences (100%) were made up of 5 (11.3%), 12 (27.3%), 19 (43.2%), and 8 (18.2%) clones from individual nematodes 1, 2, 3, and 4 designated as SSU1B, SSU12A, SSU13B, and SSU25A, respectively. Reniform nematode variant 1 (RN_VAR1) consisted of 11 (25.0%) clones out of the 44 clones sequenced and these were very similar in terms of variable sites, and consisted of 1, 4, 0, and 6 clones for SSU1B, SSU12A, SSU13B, and SSU25A, respectively. A majority of clones sequenced (33 or 75%) were classified as reniform variant 2 (RN_VAR2) sequences. These 33 clone sequences (100%) consisted of 4, 8, 19, and 2 clones for SSU1B, SSU12A, SSU13B, and SSU25A, respectively. Comparisons of the consensus sequences from the variant clones of the RN to GenBank gave varying hits to nematode sequences. Variant 1 (RN_VAR1) sequence had the top-most hit to *Rotylenchulus reniformis* isolate wb2 *18S* ribosomal RNA gene (GenBank accession number EU306342) with a 98% similarity, and a bit score of 3,125. Variant 2 (RN_VAR2) sequence had a top-most hit to RN_VAR1, followed by *Heterodera schachtii* isolate wb15 (GenBank accession number EU306355), with a 95% similarity, bit score was 2,791. The MSA analysis identified 96 (5.5%) nucleotide sites that distinguish the two *18S* rRNA variants (**Tables 2** **and** **3**). These positions were dispersed widely between 95 to 1673 bp within the *18S* rRNA gene. Seven distinct sites (0.4%) had indels within the consensus sequence at base positions 155, 156, 769, 1,330, 1,331, 1,633 and 1,634. The number of conserved regions among the two variants of the RNs was determined using default parameters [24]. Twenty-six conserved regions were present among both RN variants (**Fig. 1**). Regions 774–980 bp and 1,084–1283 bp had the highest stretch of conserved bases of 207 and 200, respectively.

**Table 1:** Oligonucleotide primers for sequencing of the RN *18S* rRNA gene.

Oligo name	Primer sequence (5′-3′)	Direction
SSUF05	GAAACTGCGAA(CT)GGCTCATT	Forward
SSUR81	TGATCC(AT) (GT)C(CT)GCAGGTTCAC	Reverse
SSUF22	TCCAAGGAAGGCAGCAGGC	Forward
SSUR13	GGGCATCACAGACCTGTTA	Reverse
T3	ATTAACCCTCACTAAAGGGA	Reverse
T7	TAATACGACTCACTATAGGG	Forward

**Table 2:** Variable sites identified among reniform nematode (RN) *18S* rRNA variants.

	Variations at specific base locations in 18S clones (15) of rRNA variant 1 (RN_VAR1)																								
	96	105	106	110	119	123	152	154	155	156	165	253	265	294	313	316	331	334	379	408	409	410	417	418	419
SSU1B8	A	G	C	T	A	T	C	C	A	T	T	G	C	C	G	C	G	C	T	G	C	T	G	A	T
SSU12A4	A	G	C	T	A	T	C	C	A	T	T	G	C	C	G	C	G	C	T	A	G	T	G	A	T
SSU12A9	A	G	C	T	A	T	C	C	A	T	T	G	C	C	G	C	G	C	T	A	G	T	G	A	T
SSU12A10	A	G	C	T	A	T	C	C	A	T	T	G	C	C	G	C	G	C	T	A	G	T	G	A	T
SSU12A12	A	G	C	T	A	T	C	C	A	T	T	G	C	C	G	C	G	C	T	A	G	T	G	A	T
SSU25A1	A	G	C	T	A	T	C	C	A	T	T	G	C	C	G	C	G	C	T	A	G	T	G	A	T
SSU25A4	A	G	C	T	A	T	C	C	A	T	T	G	C	C	G	C	G	C	T	A	G	T	G	A	T
SSU25A5	A	G	C	T	A	T	C	C	A	T	T	G	C	C	G	C	G	C	T	A	G	T	G	A	T
SSU25A6	A	G	C	T	A	T	C	C	A	T	T	G	C	C	G	C	G	C	T	G	C	T	G	A	T
SSU25A8	A	G	C	T	A	T	C	C	A	T	T	G	C	C	G	C	G	C	T	A	G	T	G	A	T
SSU25A10	A	G	C	T	A	T	C	C	A	-	T	G	C	C	G	C	G	C	T	A	C	T	G	A	T
**CONSENSUS**	**A**	**G**	**C**	**T**	**A**	**T**	**C**	**C**	**A**	**T**	**T**	**G**	**C**	**C**	**G**	**C**	**G**	**C**	**T**	**A**	**G**	**T**	**G**	**A**	**T**
	Variations at specific base locations in 18S clones (34) of rRNA variant 2 (RN_VAR2)																								
SSU1B1	C	A	A	A	G	C	T	A	–	–	A	A	T	A	A	G	C	T	G	A	C	C	A	T	C
SSU1B4	C	A	A	C	G	C	T	A	–	–	A	A	T	A	A	G	C	T	G	G	C	C	A	T	C
SSU1B5	C	A	A	C	G	C	T	A	–	–	A	A	T	A	A	G	C	T	G	A	C	C	A	T	C
SSU1B6	C	A	A	C	G	C	T	A	–	–	A	A	T	A	A	G	C	T	G	A	C	C	A	T	C
SSU12A1	C	A	A	C	G	C	T	A	–	–	A	A	T	A	A	G	C	T	G	A	C	C	A	T	C
SSU12A2	C	A	A	C	G	C	T	A	–	–	A	A	T	A	A	G	C	T	G	A	C	C	A	T	C
SSU12A3	C	A	A	C	G	C	T	A	–	–	A	A	T	A	A	G	C	T	G	A	C	C	A	T	C
SSU12A5	C	A	A	C	G	C	T	A	–	–	A	A	T	A	A	G	C	T	G	A	C	C	A	T	C
SSU12A6	C	A	A	C	G	C	T	A	–	–	A	A	T	A	A	G	C	T	G	A	C	C	A	T	C
SSU12A7	C	A	A	C	G	C	T	A	–	–	A	A	T	A	A	G	C	T	G	A	C	C	A	T	C
SSU12A8	C	A	A	C	G	C	T	A	–	–	A	A	T	A	A	G	C	T	G	A	C	C	A	T	C
SSU12A13	C	A	A	C	G	C	T	A	–	–	A	A	T	A	A	G	C	T	G	A	C	C	A	T	C
SSU25A11	C	A	A	C	G	C	T	A	–	–	A	A	T	A	A	G	C	T	G	A	C	C	A	T	C
SSU25A12	C	A	A	C	G	C	T	A	–	–	A	A	T	A	A	G	C	T	G	A	C	C	A	T	C
SSU13B1	C	A	A	C	G	C	T	A	–	–	A	A	T	A	A	G	C	T	G	A	C	C	A	T	C
SSU13B2	C	A	A	C	G	C	T	A	–	–	A	A	T	A	A	G	C	T	G	A	C	C	A	T	C
SSU13B3	C	A	A	C	G	C	T	A	–	–	A	A	T	A	A	G	C	T	G	A	C	C	A	T	C
SSU13B4	C	A	A	C	G	C	T	A	–	–	A	A	T	A	A	G	C	T	G	A	C	C	A	T	C
SSU13B5	C	A	A	C	G	C	T	A	–	–	A	A	T	A	A	G	C	T	G	A	C	C	A	T	C
SSU13B6	C	A	A	C	G	C	T	A	–	–	A	A	T	A	A	G	C	T	G	A	C	C	A	T	C
SSU13B7	C	A	A	C	G	C	T	A	–	–	A	A	T	A	A	G	C	T	G	A	C	C	A	T	C
SSU13B9	C	A	A	C	G	C	T	A	–	–	A	A	T	A	A	G	C	T	G	A	C	C	A	T	C
SSU13B10	C	A	A	C	G	C	T	A	–	–	A	A	T	A	A	G	C	T	G	A	C	C	A	T	C
SSU13B11	C	A	A	C	G	C	T	A	–	–	A	A	T	A	A	G	C	T	G	A	C	C	A	T	C
SSU13B12	C	A	A	C	G	C	T	A	–	–	A	A	T	A	A	G	C	T	G	A	C	C	A	T	C
SSU13B13	C	A	A	C	G	C	T	A	–	–	A	A	T	A	A	G	C	T	G	A	C	C	A	T	C
SSU13B14	C	A	A	C	G	C	T	A	–	–	A	A	T	A	A	G	C	T	G	A	C	C	A	T	C
SSU13B15	C	A	A	C	G	C	T	A	–	–	A	A	T	A	A	G	C	T	G	A	C	C	A	T	C
SSU13B16	C	A	A	C	G	C	T	A	–	–	A	A	T	A	A	G	C	T	G	A	C	C	A	T	C
SSU13B17	C	A	A	C	G	C	T	A	–	–	A	A	T	A	A	G	C	T	G	A	C	C	A	T	C
SSU13B18	C	A	A	C	G	C	T	A	–	–	A	A	T	A	A	G	C	T	G	A	C	C	A	T	C
SSU13B19	C	A	A	C	G	C	T	A	–	–	A	A	T	A	A	G	C	T	G	A	C	C	A	T	C
SSU13B20	C	A	A	C	G	C	T	A	–	–	A	A	T	A	A	G	C	T	G	A	C	C	A	T	C
**CONSENSUS**	**C**	**A**	**A**	**A**	**G**	**C**	**T**	**A**	–	–	**A**	**A**	**T**	**A**	**A**	**G**	**C**	**T**	**G**	**A**	**C**	**C**	**A**	**T**	**C**

427	428	449	474	514	570	571	573	575	582	587	593	594	595	603	604	606	610	619	631	636	642	657	659
A	G	C	C	G	A	G	T	T	C	T	T	G	T	T	G	G	G	–	C	T	C	A	T
A	C	C	C	G	A	G	T	T	C	T	T	G	T	T	G	A	A	–	C	T	C	A	T
A	C	C	C	G	A	G	T	T	C	T	T	G	T	T	G	A	A	–	C	T	C	A	T
A	C	C	C	G	A	G	T	T	C	T	T	G	T	T	G	A	A	–	C	T	C	A	T
A	C	C	C	G	A	G	T	T	C	T	T	G	T	T	G	A	A	–	C	T	C	A	T
A	C	C	C	G	A	G	T	T	C	T	T	G	T	T	G	A	A	–	C	T	C	A	T
A	C	C	C	G	A	G	T	T	C	T	T	G	T	T	G	A	A	–	C	T	C	A	T
A	C	C	C	G	A	G	T	T	C	T	T	G	T	T	G	A	A	–	C	T	C	A	T
A	G	C	C	G	A	G	T	T	T	T	T	A	T	T	G	G	G	–	C	T	C	A	C
A	C	C	C	G	A	G	T	T	C	T	T	G	T	T	G	A	A	–	C	T	C	A	T
A	G	C	C	G	A	G	T	T	C	T	T	G	T	T	G	A	G	–	C	T	C	A	T
**A**	**C**	**C**	**C**	**G**	**A**	**G**	**T**	**T**	**C**	**T**	**T**	**G**	**T**	**T**	**G**	**A**	**A**	–	**C**	**T**	**C**	**A**	**T**

G	G	T	G	C	G	A	C	A	T	A	C	A	C	C	T	G	G	T	T	C	T	G	C
G	G	T	G	C	G	A	C	A	T	A	C	A	C	C	T	G	G	T	T	C	T	G	C
G	G	T	G	C	G	A	C	A	T	A	C	A	C	C	T	G	G	T	T	C	T	G	C
G	G	T	G	C	G	A	C	A	T	A	C	A	C	C	T	G	G	T	T	C	T	G	C
G	G	T	G	C	G	A	C	A	T	G	C	A	C	C	T	G	G	T	T	C	T	G	C
G	G	T	G	C	G	A	C	A	T	A	C	A	C	C	T	G	G	T	T	C	T	G	C
G	G	T	G	C	G	A	C	A	T	A	C	A	C	C	T	G	G	T	T	C	T	G	C
G	G	T	G	C	G	A	C	A	T	A	C	A	C	C	T	G	G	T	T	C	T	G	C
G	G	T	G	C	G	A	C	A	T	A	C	A	C	C	T	G	G	T	T	C	T	G	C
G	G	T	G	C	G	A	C	A	T	A	C	A	C	C	T	G	G	T	T	C	T	G	C
G	G	T	G	C	G	A	C	A	T	A	C	A	C	C	T	G	G	T	T	C	T	G	C
G	G	T	G	C	G	A	C	A	T	A	C	A	C	C	T	G	G	T	T	C	T	G	C
G	G	T	G	C	G	A	C	A	T	A	C	A	C	C	T	G	G	T	T	C	T	G	C
G	G	T	G	C	G	A	C	A	T	A	C	A	C	C	T	G	G	T	T	C	T	G	C
G	G	T	G	C	G	A	C	A	T	A	C	A	C	C	T	G	G	T	T	C	T	G	C
G	G	T	G	C	G	A	C	A	T	A	C	A	C	C	T	G	G	T	T	C	T	G	C
G	G	T	G	C	G	A	C	A	T	A	C	A	C	C	T	G	G	T	T	C	T	G	C
G	G	T	G	C	G	A	C	A	T	A	C	A	C	C	T	G	G	T	T	C	T	G	C
G	G	T	G	C	G	A	C	A	T	A	C	A	C	C	T	G	G	T	T	C	T	G	C
G	G	T	G	C	G	A	C	A	T	A	C	A	C	C	T	G	G	T	T	C	T	G	C
G	G	T	G	C	G	A	C	A	T	A	C	A	C	C	T	G	G	T	T	C	T	G	C
G	G	T	G	C	G	A	C	A	T	A	C	A	C	C	T	G	G	T	T	C	T	G	C
G	G	T	G	C	G	A	C	A	T	A	C	A	C	C	T	G	G	T	T	C	T	G	C
G	G	T	G	C	G	A	C	A	T	A	C	A	C	C	T	G	G	T	T	C	T	G	C
G	G	T	G	C	G	A	C	A	T	A	C	A	C	C	T	G	G	T	T	C	T	G	C
G	G	T	G	C	G	A	C	A	T	A	C	A	C	C	T	G	G	T	T	C	T	G	C
G	G	T	G	C	G	G	C	A	T	A	C	A	C	C	T	G	G	T	T	C	T	G	C
G	G	T	G	C	G	A	C	A	T	A	C	A	C	C	T	G	G	T	T	C	T	G	C
G	G	T	G	C	G	A	C	A	T	A	C	A	C	C	T	G	G	T	T	C	T	G	C
G	G	T	G	C	G	A	C	A	T	A	C	A	C	C	T	G	G	T	T	C	T	G	C
G	G	T	G	C	G	A	C	A	T	A	C	A	C	C	T	G	G	T	T	C	T	G	C
G	G	T	G	C	G	A	C	A	T	A	C	A	C	C	T	G	G	T	T	C	T	G	C
G	G	T	G	C	G	A	C	A	T	A	C	A	C	C	T	G	G	T	T	C	T	G	C
**G**	**G**	**T**	**G**	**C**	**G**	**A**	**C**	**A**	**T**	**A**	**C**	**A**	**C**	**C**	**T**	**G**	**G**	**T**	**T**	**C**	**T**	**G**	**C**

**Table 3:** Variable sites identified among reniform nematode (RN) *18S* rRNA variants.

	Variations at specific base locations in 18S clones (15) of rRNA variant 1 (RN_VAR1)																					
	671	672	673	769	981	983	984	987	992	996	1284	1286	1300	1330	1331	1335	1411	1412	1415	1421	1428	1439
SSU1B8	A	G	C	T	A	T	C	C	–	T	A	G	C	T	T	-	C	G	A	C	G	A
SSU12A4	A	G	C	T	A	T	C	C	–	T	A	G	C	T	T	A	C	G	A	C	G	A
SSU12A9	A	G	C	T	A	T	C	C	–	T	A	G	C	T	T	A	C	G	A	C	G	A
SSU12A10	A	G	C	T	A	T	C	C	–	T	A	G	C	T	T	A	C	G	A	C	G	A
SSU12A12	A	G	C	T	A	T	C	C	–	T	A	G	C	T	T	A	C	G	A	C	G	A
SSU25A1	A	G	C	T	A	T	C	C	–	T	A	G	C	T	T	A	C	G	A	C	G	A
SSU25A4	A	G	C	T	A	T	C	C	–	T	A	G	C	T	T	A	C	G	A	C	G	A
SSU25A5	A	G	C	T	A	T	C	C	–	T	A	G	C	T	T	A	C	G	A	C	G	A
SSU25A6	A	G	C	T	A	T	C	C	–	T	A	G	C	T	T	A	C	G	A	C	G	A
SSU25A8	A	G	C	T	A	T	C	C	–	T	A	G	C	T	T	A	C	G	A	C	G	A
SSU25A10	A	G	C	T	A	T	C	C	–	T	A	G	C	T	T	A	C	G	A	T	G	A
**CONSENSUS**	**A**	**G**	**C**	**T**	**A**	**T**	**C**	**C**	–	**T**	**A**	**G**	**C**	**T**	**T**	**A**	**C**	**G**	**A**	**C**	**G**	**A**
	**Variations at specific base locations in 18S clones (34) of rRNA variant 2 (RN_VAR2)**																					
SSU1B1	G	A	G	-	G	C	G	T	T	C	G	A	T	–	–	G	A	T	G	T	C	G
SSU1B4	G	A	G	-	G	C	G	T	T	C	G	A	T	–	–	G	A	T	G	T	C	G
SSU1B5	G	A	G	-	G	C	G	T	T	C	G	A	T	–	–	G	A	T	G	T	C	G
SSU1B6	G	A	G	-	G	C	G	T	T	C	G	A	T	–	–	G	A	T	G	T	C	G
SSU12A1	G	A	G	-	G	C	G	T	T	C	G	A	T	–	–	G	A	T	G	T	C	G
SSU12A2	G	A	G	-	G	C	G	T	T	C	G	A	T	–	–	G	A	T	G	C	C	G
SSU12A3	G	A	G	-	G	C	G	T	T	C	G	A	T	–	–	G	A	T	G	C	C	G
SSU12A5	G	A	G	-	G	C	G	T	T	C	G	A	T	–	–	G	A	T	G	T	C	G
SSU12A6	G	A	G	-	G	C	G	T	T	C	G	A	T	–	–	G	A	T	G	T	C	G
SSU12A7	G	A	G	-	G	C	G	T	T	C	G	A	T	–	–	G	A	T	G	T	C	G
SSU12A8	G	A	G	-	G	C	G	T	T	C	G	A	T	–	–	G	A	T	G	T	C	G
SSU12A13	G	A	G	-	G	C	G	T	T	C	G	A	T	–	–	G	A	T	G	T	C	G
SSU25A11	G	A	G	-	G	C	G	T	T	C	G	A	T	–	–	G	A	T	G	T	C	G
SSU25A12	G	A	G	-	G	C	G	T	T	C	G	A	T	–	–	G	A	T	G	T	C	G
SSU13B1	G	A	G	-	G	C	G	T	T	C	G	A	T	–	–	G	A	T	G	T	C	G
SSU13B2	G	A	G	-	G	C	G	T	T	C	G	A	T	–	–	G	A	T	G	T	C	G
SSU13B3	G	A	G	-	G	C	G	T	T	C	G	A	T	–	–	G	A	T	G	T	C	G
SSU13B4	G	A	G	-	G	C	G	T	T	C	G	A	T	–	–	G	A	T	G	T	C	G
SSU13B5	G	A	G	-	G	C	G	T	T	C	G	A	T	–	–	G	A	T	G	T	C	G
SSU13B6	G	A	G	-	G	C	G	T	T	C	G	A	T	–	–	G	A	T	G	T	C	G
SSU13B7	G	A	G	-	G	C	G	T	T	C	G	A	T	–	–	G	A	T	G	T	C	G
SSU13B9	G	A	G	-	G	C	G	T	T	C	G	A	T	–	–	G	A	T	G	T	C	G
SSU13B10	G	A	G	-	G	C	G	T	T	C	G	A	T	–	–	G	A	T	G	T	C	G
SSU13B11	G	A	G	-	G	C	G	T	T	C	G	A	T	–	–	G	A	T	G	T	C	G
SSU13B12	G	A	G	-	G	C	G	T	T	C	G	A	T	–	–	G	A	T	G	T	C	G
SSU13B13	G	A	G	-	G	C	G	T	T	C	G	A	T	–	–	G	A	T	G	T	C	G
SSU13B14	G	A	G	-	G	C	G	T	T	C	G	A	T	–	–	G	A	T	G	T	C	G
SSU13B15	G	A	G	-	G	C	G	T	T	C	G	A	T	–	–	G	A	T	G	T	C	G
SSU13B16	G	A	G	-	G	C	G	T	T	C	G	G	T	–	–	G	A	T	G	T	C	G
SSU13B17	G	A	G	-	G	C	G	T	T	C	G	A	T	–	–	G	A	T	G	T	C	G
SSU13B18	G	A	G	-	G	C	G	T	T	C	G	A	T	–	–	G	A	T	G	T	C	G
SSU13B19	G	A	G	-	G	C	G	T	T	C	G	A	T	–	–	G	A	T	G	T	C	G
SSU13B20	G	A	G	G	G	C	G	T	T	C	G	A	T	–	–	G	A	T	G	T	C	G
**CONSENSUS**	**G**	**A**	**G**	**-**	**G**	**C**	**G**	**T**	**T**	**C**	**G**	**A**	**T**	–	–	**G**	**A**	**T**	**G**	**T**	**C**	**G**

1441	1442	1451	1452	1458	1488	1510	1518	1540	1599	1616	1623	1627	1633	1634	1635	1636	1642	1643	1646	1651	1653	1655	1668	1672
C	G	C	C	T	C	T	G	C	A	A	A	A	T	T	C	G	C	G	G	T	A	A	T	A
C	G	C	C	T	C	T	G	C	A	A	A	A	T	T	C	G	C	G	G	T	A	A	T	A
C	G	C	C	T	C	T	G	C	A	A	A	A	T	T	C	G	C	G	G	T	A	A	T	A
C	G	C	C	T	C	T	G	C	A	A	A	A	T	T	C	G	C	G	G	T	A	A	-	A
C	G	C	C	T	C	T	G	C	A	A	A	A	T	T	C	G	C	G	G	T	A	A	T	A
C	G	C	C	T	C	T	G	C	A	A	A	A	T	T	C	G	C	G	G	T	A	A	T	A
C	G	C	C	T	C	T	G	C	A	A	A	A	T	T	C	G	C	G	G	T	A	A	T	A
C	G	C	C	T	C	T	G	C	A	A	A	A	T	T	C	G	C	G	G	T	A	A	T	A
C	G	C	C	T	C	T	G	C	A	A	A	G	T	A	C	G	C	G	G	T	G	A	T	A
C	G	C	C	T	C	T	G	C	A	A	A	A	T	T	C	G	C	G	G	T	A	A	T	A
C	G	C	A	T	C	T	G	C	A	A	A	A	T	T	C	G	C	G	G	T	A	A	T	A
**C**	**G**	**C**	**A**	**T**	**C**	**T**	**G**	**C**	**A**	**A**	**A**	**A**	**T**	**T**	**C**	**G**	**C**	**G**	**G**	**T**	**A**	**A**	**T**	**A**

T	T	G	A	A	T	C	A	T	G	T	G	G	–	–	T	C	G	T	A	A	G	G	C	C
T	T	G	A	A	T	C	A	T	G	T	G	G	–	–	T	C	G	T	A	A	G	G	C	C
T	T	G	A	A	T	C	A	T	G	T	G	G	–	–	T	C	G	T	A	A	G	G	C	C
T	T	G	A	A	T	C	A	T	G	T	G	G	–	–	T	C	G	T	A	A	G	G	C	C
T	T	G	A	A	T	C	A	T	G	T	G	G	–	–	T	C	G	T	A	A	G	G	C	C
T	T	G	A	A	T	C	A	T	G	T	G	G	–	–	T	C	G	T	A	A	G	G	C	C
T	T	G	A	A	T	C	A	T	G	T	G	G	–	–	T	C	G	T	A	A	G	G	C	C
T	T	G	A	A	T	C	A	T	G	T	G	G	–	–	T	C	G	T	A	A	G	G	C	C
T	T	G	A	A	T	C	A	T	G	T	G	G	–	–	T	C	G	T	A	A	G	G	C	C
T	T	G	A	A	T	C	A	T	G	T	G	G	–	–	T	C	G	T	A	A	G	G	C	C
T	T	G	A	A	T	C	A	T	G	T	G	G	–	–	T	C	G	T	A	A	G	G	C	C
T	T	G	A	A	T	C	A	T	G	T	G	G	–	–	T	C	G	T	A	A	G	G	C	C
T	T	G	A	A	T	C	A	T	G	T	G	G	–	–	T	C	G	T	A	A	G	G	C	C
T	T	G	A	A	T	C	A	T	G	T	G	G	–	–	T	C	G	T	A	A	G	G	C	C
T	T	G	A	A	T	C	A	T	G	T	G	G	–	–	T	C	G	T	A	A	G	G	C	C
T	T	G	A	A	T	C	A	T	G	T	G	G	–	–	T	C	G	T	A	A	G	G	C	C
T	T	G	A	A	T	C	A	T	G	T	G	G	–	–	T	C	G	T	A	A	G	G	C	C
T	T	G	A	A	T	C	A	T	G	T	G	G	–	–	T	C	G	T	A	A	G	G	C	C
T	T	G	A	A	T	C	A	T	G	T	G	G	–	–	T	C	G	T	A	A	G	G	C	C
T	T	G	A	A	T	C	A	T	G	T	G	G	–	–	T	C	G	T	A	A	G	G	C	C
T	T	G	A	A	T	C	A	T	G	T	G	G	–	–	T	C	G	T	A	A	G	G	C	C
T	T	G	A	A	T	C	A	T	G	T	G	G	–	–	T	C	G	T	A	A	G	G	C	C
T	T	G	A	A	T	C	A	T	G	T	G	G	–	–	T	C	G	T	A	A	G	G	C	C
T	T	G	A	A	T	C	A	T	G	T	G	G	–	–	T	C	G	T	A	A	G	G	C	C
T	T	G	A	A	T	C	A	T	G	T	G	G	–	–	T	C	G	T	A	A	G	G	C	C
T	T	G	A	A	T	C	A	T	G	T	G	G	–	–	T	C	G	T	A	A	G	G	C	C
T	T	G	A	A	T	C	A	T	G	T	G	G	–	–	T	C	G	T	A	A	G	G	C	C
T	T	G	A	A	T	C	A	T	G	T	G	G	–	–	T	C	G	T	A	A	G	G	C	C
T	T	G	A	A	T	C	A	T	G	T	G	G	–	–	T	C	G	T	A	A	G	G	C	C
T	T	G	A	A	T	C	A	T	G	T	G	G	–	–	T	C	G	T	A	A	G	G	C	C
T	T	G	A	A	T	C	A	T	G	T	G	G	–	–	T	C	G	T	A	A	G	G	C	C
T	T	G	A	A	T	C	A	T	G	T	G	G	–	–	T	C	G	T	A	A	G	G	C	C
T	T	G	A	A	T	C	A	T	G	T	G	G	–	–	T	C	G	T	A	A	G	G	C	C
**T**	**T**	**G**	**A**	**A**	**T**	**C**	**A**	**T**	**G**	**T**	**G**	**G**	–	–	**T**	**C**	**G**	**T**	**A**	**A**	**G**	**G**	**C**	**C**

**Figure 2:**
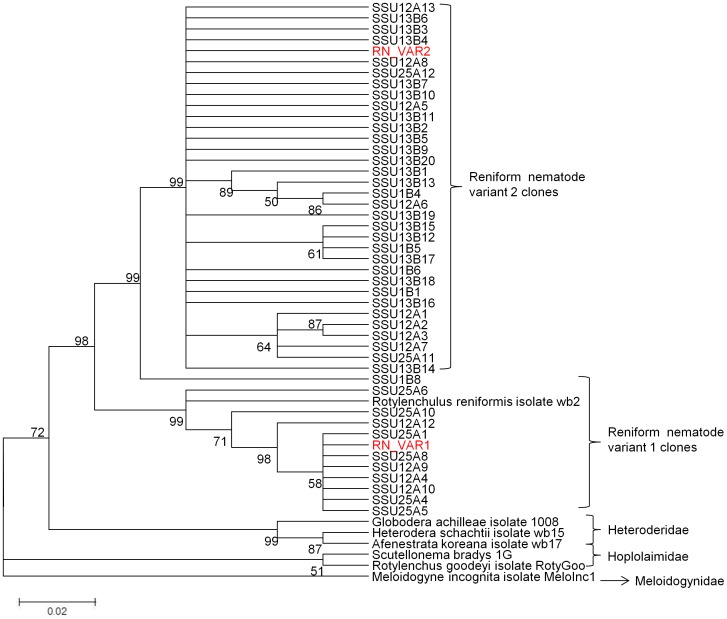
Neighbor-Joining (NJ) analyses for 44 RN *18S* rRNA clones and nematodes in the super-families, Hoplolaimidae, Heteroderidae, Rotylenchulidae, and Meloidogynidae.

**Figure 3:**
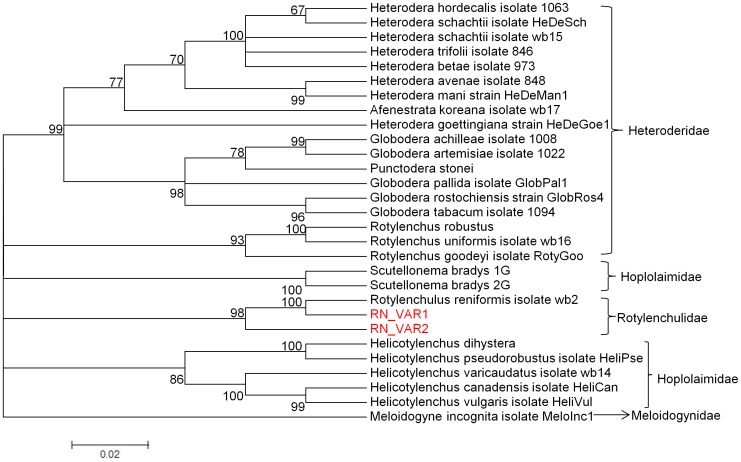
Neighbor-Joining (NJ) analysis for RN variants (RN_VAR1 and RN_VAR2) and nematodes in the super-families, Hoplolaimidae, Heteroderidae, Rotylenchulidae, and Meloidogynidae.

Initial phylogenetic analysis was based on 44 RN clone sequences, RN_VAR1, RN_VAR2 consensus sequences, two sequences each from a taxa in the super-family Heteroderidae (*Afenestrata koreana* isolate wb17, and *Globodera achilleae* isolate 1008), Hoplolaimidae (*Scutellonema bradys* 1G and *Rotylenchus goodeyi* isolate RotyGoo), and one sequence each from Rotylenchulidae (*Rotylenchulus reniformis* isolate wb2), and Meloidogynidae super-family *Meloidogyne incognita* isolate MeloInc1 (**Fig. 2**). A neighbor-joining (NJ) tree was generated with bootstrap test of phylogeny with 10,000 replications, random seed of 64,238 model used was nucleotide: maximum composite likelihood, substitutions (d:transitions + transversions), pattern among lineages was homogeneous, rates among sites were uniform, Gaps/missing data- complete deletion. The NJ tree showed a major split between clones consisting of RN_VAR1, and RN_VAR2. Other nematodes belonging to the various super-families clustered together. *R. reniformis* isolate wb2 grouped with RN_VAR1 and *M. incognita* isolate MeloInc1 did not group with any other taxa (**Fig. 2**). Bootstrapping using the NJ clearly separated the clones (SSU12A, SSU25A, SSU1B, and SSU13B). The inclusion of the consensus sequences from these clones, thus (RN_VAR1 and RN_VAR2) in the NJ tree resulted in these clustering with their resultant clone sequences. Interestingly, clones from nematode isolate 13B were of RN_VAR2, however, clones from the other three nematodes were either of RN_VAR1 or RN_VAR2 and clustered as such. A second phylogram was generated using a NJ tree, with the same parameters as the first. Nematodes belonging to the super-families Heteroderidae, Hoplolaimidae, Rotylenchulidae and Meloidogynidae were used in the analysis (**Table 4**). The NJ tree showed taxa belonging to the same super-families being clustered together. The RN_VAR1 grouped with *Rotylenchulus reniformis* isolate wb2 with a bootstrap percentage of 100, and *Meloidogyne incognita* isolate MeloInc1, the out-group was not closely related to any other taxa (**Fig. 3**).

**Table 4:** Nematode species belonging to Tylenchoidea, GenBank accession numbers, and length of *18S* rRNA sequence.

Super-family	Nematodes	GenBank No.	Sequence length (bp)
Heteroderidae	*Heterodera avenae* isolate 848	FJ040403	1,725
	*Heterodera betae* isolate 973	FJ040404	1,725
	*Heterodera goettingiana* strain HeDeGoe1	EU669915	1,725
	*Heterodera hordecalis* isolate 1063	FJ040405	1,725
	*Heterodera mani* strain HeDeMan1	EU669916	1,725
	*Heterodera schachtii* isolate HeDeSch	AY284617	1,744
	*Heterodera schachtii isolate wb15*	EU306355	1,772
	*Heterodera trifolii* isolate 846	FJ040402	1,724
	*Afenestrata koreana* isolate wb17	EU306357	1,788
	*Globodera achilleae* isolate 1008	FJ040399	1,726
	*Globodera artemisiae* isolate 1022	FJ040400	1,726
	*Globodera pallida* isolate GlobPal1	AY284618	1,746
	*Globodera rostochiensis* strain GlobRos4	AY593880	1,728
	*Globodera tabacum* isolate GlobTab4	FJ040401	1,726
	*Punctodera stonei*	EU682391	1,727
Hoplolaimidae	*Rotylenchus goodeyi* isolate RotyGoo	AY284609	1,634
	*Rotylenchus robustus*	AJ966503	1,796
	*Rotylenchus uniformis* isolate wb16	EU306356	1,796
	*Scutellonema bradys* 1G	AY271723	1,811
	*Scutellonema bradys* 2G	AJ966504	1,803
	*Helicotylenchus Canadensis* isolate HeliCan	AY284605	1,635
	*Helicotylenchus dihystera*	AJ966486	1,790
	*Helicotylenchus pseudorobustus* isolate HeliPse	AY284606	1,755
	*Helicotylenchus varicaudatus* isolate wb14	EU306354	1,802
	*Helicotylenchus vulgaris* isolate HeliVul	AY284607	1,751
Rotylenchulidae	*Rotylenchulus reniformis* isolate wb2	EU306342	1,873
Meloidogynidae	*Meloidogyne incognita* isolate MeloInc1	AY284621	1,730

**Figure 4:**
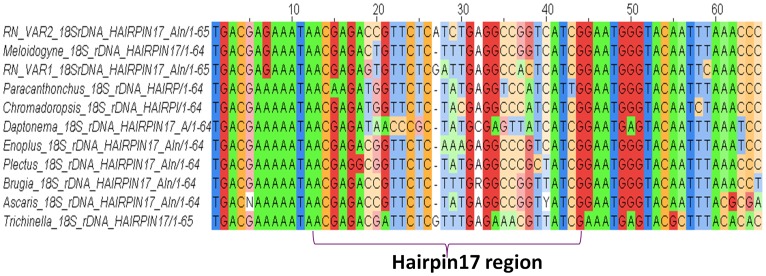
Multiple sequence alignment of the primary structure of the 18S rRNA gene in RN showing hairpin 17 corresponding to positions 391 - 455 in RN_VAR1 and RN_VAR2.

**Figure 5:**
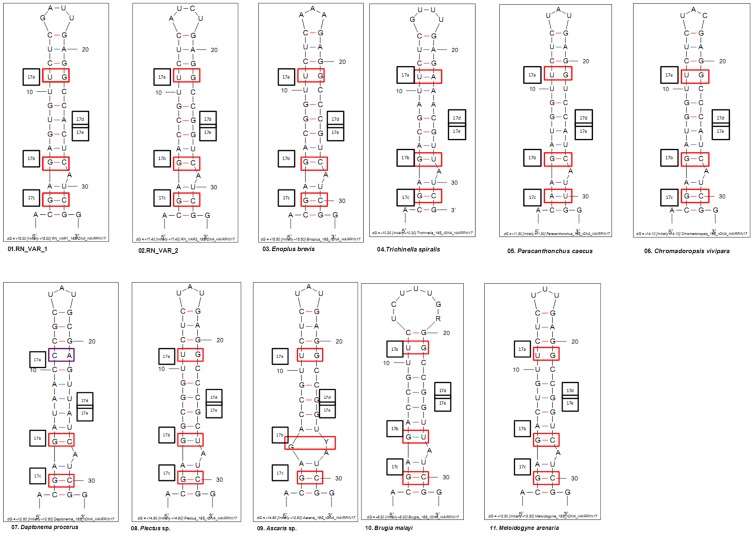
The secondary structure of hairpin 17 of the 18S rDNA for RN_VAR1, RN_VAR2 of RN and 9 other nematode species. The pairs of nucleotides peculiar to certain taxa are boxed in red at positions 17a, 17b, and 17c. Base pair for *Daptonema,* CA at position 17a is boxed in violet color.

BLASTN results of RN_VAR1 and RN_VAR2 to REPLIg genomic library gave hits of 83 (26%) and 236 (74%), respectively. The average alignment length and bit scores for both variants were 400.43 bp and 749.64 for RN_VAR1 and 441.40 bp and 832.79 for RN_VAR2, respectively. Comparison of our RN *18S* rDNA variant sequences to our genomic sequences to determine the presence of these variants in the RN genome, showed RN_VAR2 with high unique hits, average % identity, and low mis-matches within the REPLIg genomic sequences (**Table 5**). Similarly, both RN_VAR1 and RN_VAR2 had 250 hits to our RN ESTs, respectively. Among these hits, 40(16%), 42(17%), 23(9%), and 145 (58%) had 0, 1, 2, and 3–21 mis-matches to the RN ESTs for RN_VAR1 respectively. For RN_VAR2, 111(44%), 88(35%), 35(14%), and 16(7%) had 0, 1, 2, and 3–5 mis-matches, respectively. The lesser number of mis-matches were observed for RN_VAR2. The secondary structures generated for RN_VAR1 and RN_VAR2 show 12 common sub-structures among these two variants (**Figs. S1 and S2**). These variants also show two and three sub-structures that were unrelated among RN_VAR1 and RN_VAR2 respectively. Sixty-three sub-structures were used in the generation of this folding pattern from 1,027 matched nucleotides. Multiple alignments of the RNA molecules showed structurally conserved areas among the RN variants and 110 single nucleotide polymorphisms (SNPs) (**Fig. S3**). The consensus structure obtained for RN_VAR1 and RN_VAR2 showed the most-likely structure both variants form together (**Fig. S4**). Multiple sequence alignment of 18S rDNA between the RN variants (VAR 1 and VAR 2) with 9 other nematodes provided evidence of the presence of hairpin 17 in the RN sequences on positions 391–455 (**Fig. 4**). Folding patterns of hairpin 17 in these nematodes reflects the conservative nature of this region (**Fig. 5**). Base pairs at positions 17a, 17b, and 17c were conserved among the RN_VAR1 and RN_VAR2 and in the other nine nematodes, and substitutions were noted at positions 17d and 17e.

**Table 5:** Blast hits among RN *18S* rRNA variants, and REPLIg 454 genomic library.

Query and Subject	Total number of unique hits	Average % ID	Average alignment length	Average mis-matches	Average number of gaps	Average bit score
RN_VAR1 vs REPLIg genomic sequences	83.00	98.97	400.43	2.23	1.55	749.64
RN_VAR2 vs REPLIg genomic sequences	236.00	99.25	441.40	1.07	2.08	832.79

## Discussion

Ribosomal DNA (rDNA) codes for RNA molecules and these are separated by intergenic spacers that control transcription of the rRNA genes. Both the intergenic spacers and the transcribed regions of the rRNA gene show evidence of greater divergence *between* and considerably less *within* a species, a phenomenon known as the concerted evolution. The *18S* rRNA gene regions of the rDNA evolve differentially and this attribute has been successfully used in phylogenetic analysis in kingdoms, phyla, classes, and orders [23], [25]. This high copy number gene of eukaryotic nuclear rDNA is generally tandemly organized on a chromosome. The gene region is divided into domains that are differentially conserved. Expansion segments evolve at high rates and are relatively more variable, and differences have been observed within the *18S* rRNA gene, due to the presence of variable regions which expand within this region [26]. The comparative analysis of 44 newly generated near full-length *18S* rDNA sequences from four female RNs distinctly show the presence of two sequence variants (RN_VAR1 and RN_VAR2). The presence of intra-individual variation within the *18S* rDNA of animal genomes is rare. Variation occurring within the *18S* rDNA of a metazoan species was first reported for *Dugesia* (*Schmidtea*) *mediterranea* which is a free-living platyhelminth. Two types of rDNA (type I and II) were observed in the genome of this organism, with 8% of nucleotides distinguishing the variants [2]. Variation has also been observed in the *18S* rDNA region of *Strongyloides* where four hyper-variable regions (HVR-I to IV) were identified within the *18S* rDNA among 34 isolates from 15 species [27]. Hyper-variable regions I to III differed from IV because of nucleotide lengths which were unequal, e.g., HVR-IV region ranged from 23 to 39 nucleotides. Similar to our study, these variable regions could serve as markers for identification of *Strongyloides* species. A study conducted using the rRNA gene loci of 12 *Drosophila* species showed the presence of variants within 7.8–8.2 kb region [9]. A range of 3 to 18 variants were present in more than 3% of the rDNA region in 11 species. Unlike our study, which focuses exclusively on the coding region, in *Drosophila* the non-coding regions had more variants than in the coding regions, and these variants were 3 to 8 times more prevalent in the expansion region. Our ongoing work also suggests that the presence of coding region variation seems to occur in individually-sequenced single nematodes from locations across Alabama and Mississippi. Variants have also been observed in the *Plasmodium* within its life cycle stages [28] and suggestion have been made that such sequence variation may impart some advantage to the host in its ability to escape susceptibility based on such genetic change. Additionally, in freshwater planarians variation has been reported in 2.5% to 3.9% within the *18S* rDNA region of various species and genera [29].

A phenomenon of the *18S* rRNA gene is the presence of slower evolving areas ‘core regions’ and ‘expansion regions’. The former is involved in substrate binding, while the expansion region is a variable region [30], [31]. Thus, the nucleotide sites distinguishing our RN *18S* rRNA variants could possibly occur within both the core and expansion regions. The specific nature of these sites may have functional roles within the *18S* rRNA genes of this nematode. An interesting question is why does this sequence variation exist? Does this variation provide an evolutionary advantage to this organism? Some of the possible explanations can only be proposed. Models used in clarifying this concern relating to concerted evolution occurring within the rDNA locus have focused on unequal crossovers and gene conversions. Unequal crossover among sister chromatids has been known to occur more frequently as compared to exchanges between homologous chromosomes [32], [33]and this may have contributed to the emergence rDNA variants observed in this study. Phylogenetic analysis provided additional clarity to the distinct grouping of clones that clustered together with their respective variant when *Meloidogyne incognita* isolate MeloInc1 was included as an outgroup branched away from the other RN clones and other taxa The second phylogram generated had inclusion of 26 other taxa belonging to the superfamily Tylenchoidea, in an attempt to place these RN variants within this superfamily. The NJ tree showed that RN_VAR1 clustered with *Rotylenchulus reniformis* isolate wb2 with a bootstrap value of 100 while the RN_VAR2 was less similar to wb2. Presence of long expansion segments in freshwater crustaceans such as *Daphnia* have been utilized to study rDNA evolution and they have sequences that maintain their function and they evolve in unison. These organisms have regions or expansion segments where sequence variations are most prevalent [34]. Compared *to Daphnia*, our study focused on the whole coding region of *18S* gene and not only on the expansion helices. The base variations were observed mainly at 96 nucleotide positions and these variations were uniformly distributed throughout the entire 18S rDNA coding region (95 to 1673 bp). This study and our ongoing research suggest that the two variants observed here are very likely ancient polymorphisms that have not escaped concerted evolution of this crucial rDNA multi-gene family. The natural question that arises with the presence of rDNA variants is whether the secondary structure is conserved and functional in a compensated manner, and does a helical structure exist in both variants of RN, or does it cause the secondary structure in the variants to become uncompensated and destabilized? The hairpin 17 region though highly conserved is still prone to substitutions which are retained by the 18S rDNA. Secondary structure analysis among Strongylida and Rhabditida distinctly revealed substitutions at positions 17a, 17b and 17c [35] The hairpin 17 secondary structures from nematodes studied showed conservative pairs UG in position 17a, GC in position 17b, and GC in position 17c, and the loss of substitutions in position17e. These substitutions were categorized as non-compensated because there were base pair losses at these positions. In our study however, the substitutions observed in positions 17d and 17e of RN_VAR1 and RN_VAR2 show compensated changes in nucleotide pairs in the conservative region of the hairpin 17 region. Our secondary structure RNA analysis showed that the SNPs among the two RN variants are compensatory. A possible explanation of two main types of sequence variants within an individual 18S gene is the presence of two locations of rDNA or nucleolar organizing regions, with occasional crossing-over between these two regions. This may be the reason why small gene conversion tracts have existed for the RN 18S rDNA. The conserved nature of nucleotides in helices or loops demonstrates a link between rRNA folding and the ribosome function [35]. As long as both sequences fold into stable secondary structures, and do not interfere with ribosome function, one of the two variants may not be eliminated. Studies involving diverging rDNA copies together with their expression potential have been reported [2], [36]. In our study it is clear that the two variants reside in the same nematode, and both *18S* variants are expressed because their sequences were present in our RN ESTs. This gene will also be useful for genetic diversity studies in the various genera of nematodes.

In summary, we have identified two distinct variants within the *18S* rRNA gene within three individual RNs, with one variant sequence being the most similar to the *Rotylenchulus reniformis* isolate wb2 (RN_VAR1). This variation may be as a result of recombination events during unequal crossover occurring between sister chromatids, or between X and Y rDNA leading to the co-evolution of the rDNA sequences on these chromosomes. Our findings can be placed in the context of earlier molecular mechanisms and paralogous or ancestral (plesiomorphic) explanations proposed by other investigators. The role of gene conversion and unequal crossover mechanisms in RN needs to be further explored to fully understand the existence of these *18S* variants. Short oligonucleotide probes designed from RN_VAR1 would yield specific markers that will differentiate this species from closely related taxa belonging to super families such as Heteroderidae, Hoplolaimidae, Rotylenchulidae, and Hoplolaimidae. In diagnostic research and environmental monitoring, probes to be developed will provide opportunities for the rapid diagnostic detection and quantification of RN compositions in environmental samples, through real-time PCR detections.. Although this study did not investigate location-specific differences in the presence of the different sequence variants, it would be interesting in the future to determine the geographic distribution of RN variants 1 and 2. This could be achieved using the full length 18S rDNA analysis of RNs collected from Africa, and other centers of RN diversity (e.g., Japan, Hawaii, and Southern half of U.S.).

## Materials and Methods

### Materials Statement

No specific permits were required for the described field studies. This is because soil samples were obtained from a single location within the state at Alabama Agricultural Research Station, BelleMina. This field is not privately owned or protected in any way, and the field studies did not involve endangered or protected species as determined by our soil diagnostic. It is regularly monitored by a Nematologist who insures that this area is extensively infested by RN.

### Nematode Extraction from the Soil and DNA Extractions

Each soil sample was thoroughly mixed and kept at 4°C; a 150 cm^3^ subsample was used for the extraction of the nematodes as described [37]. These populations were further maintained on cotton cultivar ‘Delta and Pineland 425 BG/RR’ (DPL 425) in the greenhouse to grow and multiply. Extracted RN suspension was placed into a petri dish using a pipette, and placed on an Olympus SZH-ILLD dissection microscope (Olympus optical Co. Ltd. Japan) using a low magnification of 10. Individual nematodes were picked using a sterilized hook and then placed in a drop of water on a glass slide for morphological identification, under an Olympus IMT-2 compound microscope (Olympus optical Co. Ltd. Japan). The identified individual nematodes were then hand-picked and immediately placed into DNA lysis buffer. DNA was extracted separately from each of the four individual female RNs using a DNeasy Blood and Tissue Kit (Qiagen, Inc., Valencia, CA) according to the manufacturer’s protocol.

### Polymerase Chain Reaction (PCR)

Extracted DNA (2.0 µl) of approximately 1ng/µl from single female RNs was transferred into PCR tubes containing 2.5 µl 10x High Fidelity PCR buffer, 1.0 µl MgCl_2_ (50 mM), 0.5 µl dNTPs (10 mM), 0.5 µl of forward and reverse primers each (10 µm) (synthesized by MWG-Biotech AG), 0.2 µl of high fidelity platinum taq (Invitrogen, Carlsbad, CA) and dNASE-free water added to a final volume of 25 µl. Primer pairs SSUR81 and SSUF05 (**Table 1**) were used in amplifying an approximately 1800 bp region of the *18S* rRNA gene of four individual female RNs separately. Both primers SSUF05 and SSUR81 had some degeneracy in their bases. PCR was performed in a Peltier Thermal Cycler (PTC) tetrad 2 DNA engine (Bio-Rad, Hercules, CA): 94°C for 2 min, then 39 cycles of: 94°C for 15 sec, 55°C for 30 sec, and 68°C for 1 min and a final extension phase of 68°C for 7 min. The quality of PCR products was checked by electrophoresis of 6 µl of PCR reaction in 1% agarose gel with ethidium bromide staining. The bands were visualized and photographed under uv light Bio-Rad gel imager (Bio-Rad, CA). The PCR product size was determined by comparing with a 1 kb DNA marker.

### Cloning of PCR Products

Polymerase Chain Reaction (PCR) products from four individual female nematodes were purified using a QIAquick PCR Purification Kit (Qiagen, Inc., Valencia, CA) according to the manufactures protocol. The fragments were then cloned into a plasmid vector using TOPO TA Cloning Kit (Invitrogen, Carlsbad, CA). The ligation reaction was made up of 4 µl of PCR product, 1 µl of salt solution (1.2 M NaCl and 0.06 M MgCl_2_), and 1 µl of TOPO vector. Several clones were picked for verification of inserts from PCR amplifications for each nematode. This was performed by amplification of the clonal DNA using M13 forward (5′-TGTAAAACGACGGCCAGT-3′) and reverse (5′-AGCGGATAACAATTTCACAC-3′) primers. PCR conditions were as follows: 94°C for 5 min, then 40 cycles of the following: 94°C for 30 sec, 55°C for 1 min, and 72°C for 1 min. The final extension phase was 72°C for 10 min. Individual bacterial colonies with inserts were picked and placed in separate 1.5 ml centrifuge tubes with 3 ml of liquid Luria- Bertani (LB ) media containing 100 µg/ml ampicillin and shaken at 37 ^○^C for 24 hours at 100 rpm in an Innova 4300 rotary incubator shaker (New Brunswick Scientific, Edison, NJ). Tubes containing bacterial cells were centrifuged for 3 min at 13,000 rpm in a Hermle MR-2 (National Labnet Company, Woodbridge, NJ) tabletop centrifuge to obtain a cell pellet. Plasmid DNA was isolated using a QIAprep Mini-prep kit (Qiagen, Inc., Valencia, CA) according to the manufacturer’s protocol.

### Sequencing

The *18S* rRNA gene of the RN was sequenced using six primers (**Table 1**). Plasmid inserts from at least ten colonies originating from each of the four nematodes were sequenced using the ABI PRISM Big Dye Terminator cycle sequencing ready reaction kit in an ABI 3700 nucleotide sequencer (Applied Biosystems, Foster City, CA) and screened for homology to nematoda sequences through BLASTN on the NCBI website (http://www.ncbi.nlm.nih.gov/Blast.cgi).

### Alignment and Phylogenetic Analysis

The SeqMan Pro within the DNASTAR Lasergene v8.0 software (DNASTAR Inc., Madison, WI) was used in generating consensus sequences and in trimming extraneous sequences outside the respective amplification fragments. Individual clone sequences from each nematode were used for multiple sequence alignment (http://align.genome.jp/ ) using default parameters and viewed using BioEdit software [24]. All assembled *18S* rRNA sequences have been deposited in GenBank under accession numbers JX406335–JX406383. Phylogenetic analyses were conducted using Molecular Evolutionary Genetics Analysis (MEGA) software version 4.0 [38].

### Sequence Examination for Chimera Presence

Potential production of chimeric DNA in our SSU rDNA clones was examined for all 49 clones. Analysis was carried out using Ballerophon (http://comp-bio.anu.edu.au/bellerophon/bellerophon.pl) with default setting (200 bp window) [39].

### Secondary Structure Analysis

The ExpaRNA tool was used in comparative structural RNA analysis to compute the best arrangement of sequence-structure motifs common among the RN variants. The LocARNA tool was also used for multiple sequence alignment of RNA molecules from the RN variants and for the generation of a consensus structure [40], [41]. The common eukaryotic structure based on the known secondary structures of 9 other nematodes species were obtained from GenBank: *Paracanthonchus caecus* (AF047888), *Daptonema procerus* (AF047889), *Chromadoropsis vivipara* (AF047891), *Enoplus brevis* (U88336), *Trichinella spiralis* (U60231), *Plectus* sp., (U61761), *Ascaris* sp. (M58348, etc.); *Brugia malayi* (H30951, etc.), and *Meloidogyne arenaria* (U42342) and aligned together with RN_VAR1 and RN_VAR2 sequences. The secondary structures of hairpin 17 were then analyzed using the mfold program with set parameters.

### Comparison of RN *18S* rRNA variants to RN Genomic and EST Sequences

The RN variants were also used in performing blasts searches (BLASTN) against unassembled reniform genomic sequences (**Table 5**). Whole Genome Amplification (WGA) was performed using a REPLI-g midi kits (Qiagen, MD), for generating a RN genomic library used in 454 sequencing. Large-scale 454 sequencing of RN genome gave a total of 732 MB for the REPLIg genomic library prepared from DNA of four individual adult female RNs pooled together. Detailed blast hits are presented in the supplemental tables between REPLIg genomic sequences andRN_VAR1 (**Table S2**), and RN_VAR2 (**Table S3)**. These RN raw reads have been deposited under the sequence read achieve (SRA) section of GenBank with accession numbers SRX099033. Furthermore, comparative analysis of the RN variant sequences to our RN ESTs generated from pooled eggs and vermiform stage cDNA libraries was undertaken. Over 50,000 sequence reads were generated from the RN transcriptome, resulting in more than 4 Mb (4,781,676 bases) of sequence data. Detailed blast hits are presented in the supplemental tables between RN ESTs and RN_VAR1 (**Table S4**), and RN_VAR2 (**Table S5)**. The ESTs have been submitted to GenBank under the accession numbers SRX098224 and SRX098225.

## Supporting Information

Figure S1 **Predicted secondary structure of 18S rDNA for RN_VAR1.** Numbers indicate similar sub-structures among RN_VAR1 and RN_VAR2, and arrows indicate dissimilar sub-structures among RN_VAR1 and RN_VAR2.
(TIF)

Figure S2 **Predicted secondary structure of 18S rDNA for RN_VAR2.** Numbers indicate similar sub-structures among RN_VAR2 and RN_VAR1, and arrows indicate dissimilar sub-structures among RN_VAR2 and RN_VAR1.
(TIF)

Figure S3 **Multiple alignments of the 18S rDNA for RN_VAR1 and RN_VAR2.** The bases highlighted in red indicate highly conserved areas between RN_VAR1 and RN_VAR2. The grooves in the grey bar represent dissimilarity among the nucleotides.
(TIF)

Figure S4 **The consensus structure formed both by RN_VAR1 and RN_VAR2 together.**
(TIF)

Table S1 **Multiple sequence alignment of 44 18S rDNA sequences from four female reniform nematodes.**
(XLSX)

Table S2 **Blast hits among RN_VAR1 and REPLIg genomic sequences.**
(XLSX)

Table S3 **Blast hits among RN_VAR2 and REPLIg genomic sequences.**
(XLSX)

Table S4 **Blast hits among RN_VAR1 and RN ESTs**
(XLSX)

Table S5 **Blast hits among RN_VAR2 and RN ESTs.**
(XLSX)
