# Supplementary figures and images for: Characterization of the Two Intra-Individual Sequence Variants in the 18S rRNA Gene in the Plant Parasitic Nematode, Rotylenchulus reniformis
Source: PLoS One. 2013 Apr 11;8(4):e60891. doi: 10.1371/journal.pone.0060891 (PMC3623918; doi:10.1371/journal.pone.0060891)

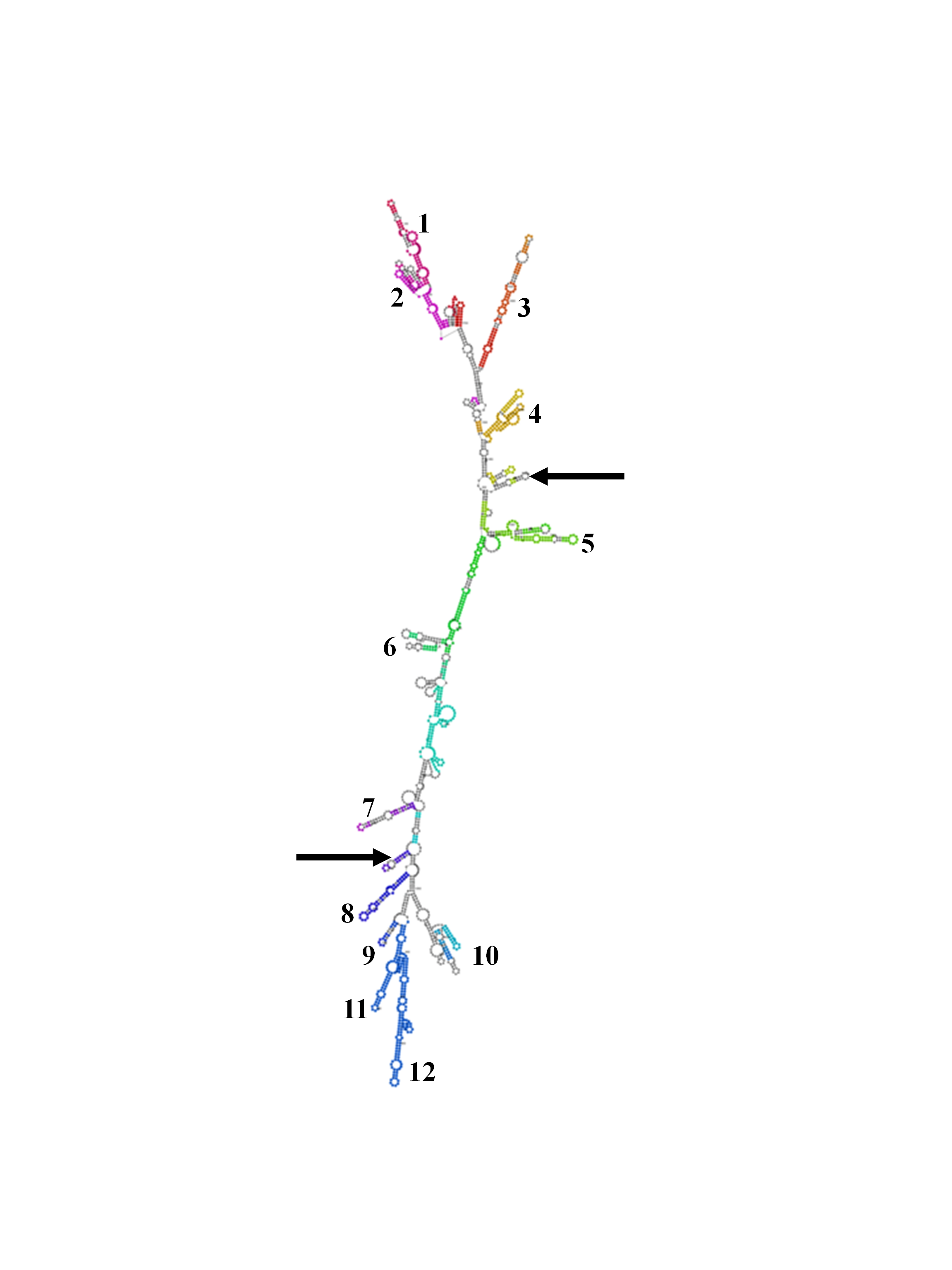

Supplement: Figure S1 — Predicted secondary structure of 18S rDNA for RN_VAR1. Numbers indicate similar sub-structures among RN_VAR1 and RN_VAR2, and arrows indicate dissimilar sub-structures among RN_VAR1 and RN_VAR2. (TIF) [file pone.0060891.s001.tif]

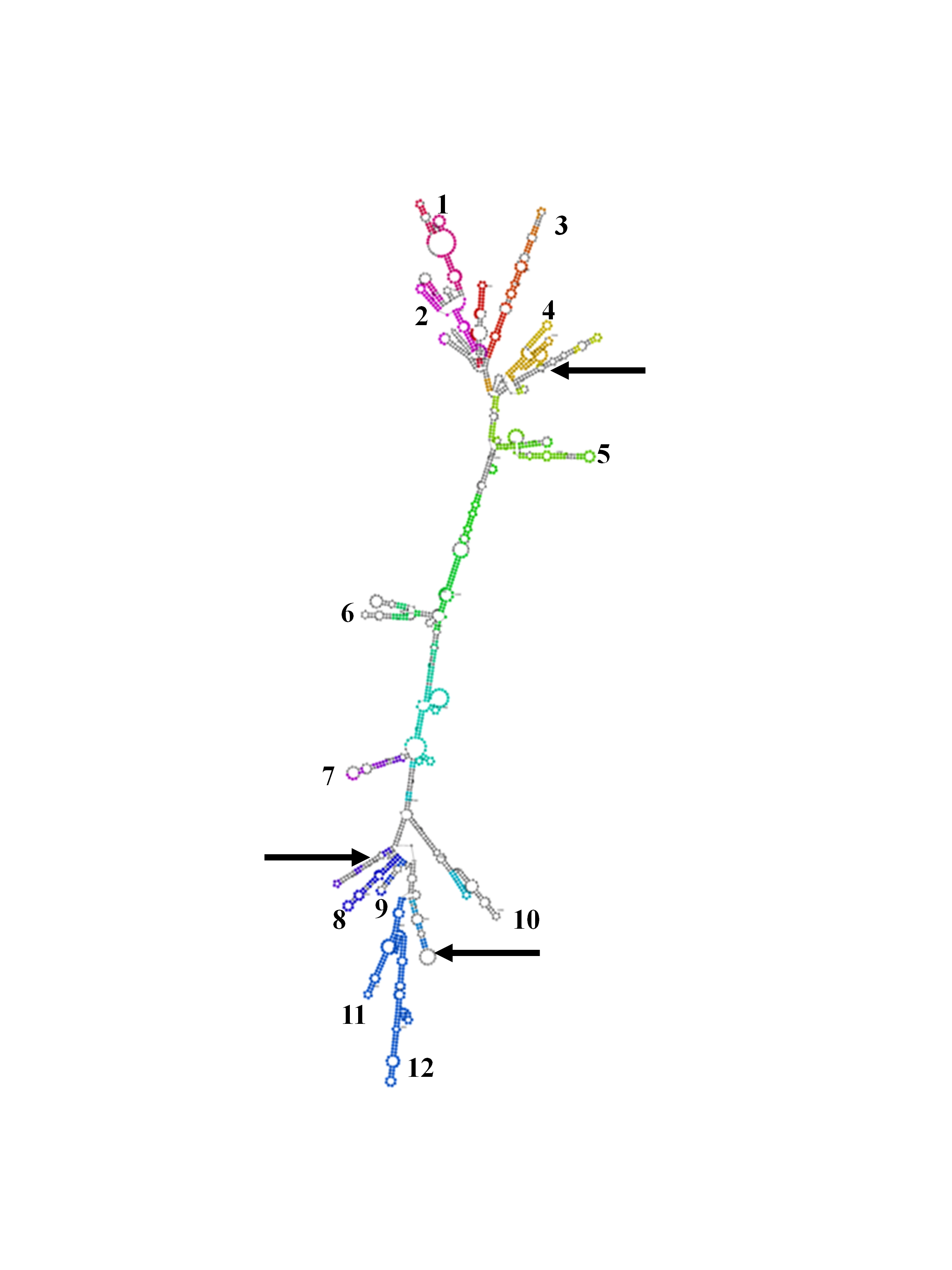

Supplement: Figure S2 — Predicted secondary structure of 18S rDNA for RN_VAR2. Numbers indicate similar sub-structures among RN_VAR2 and RN_VAR1, and arrows indicate dissimilar sub-structures among RN_VAR2 and RN_VAR1. (TIF) [file pone.0060891.s002.tif]

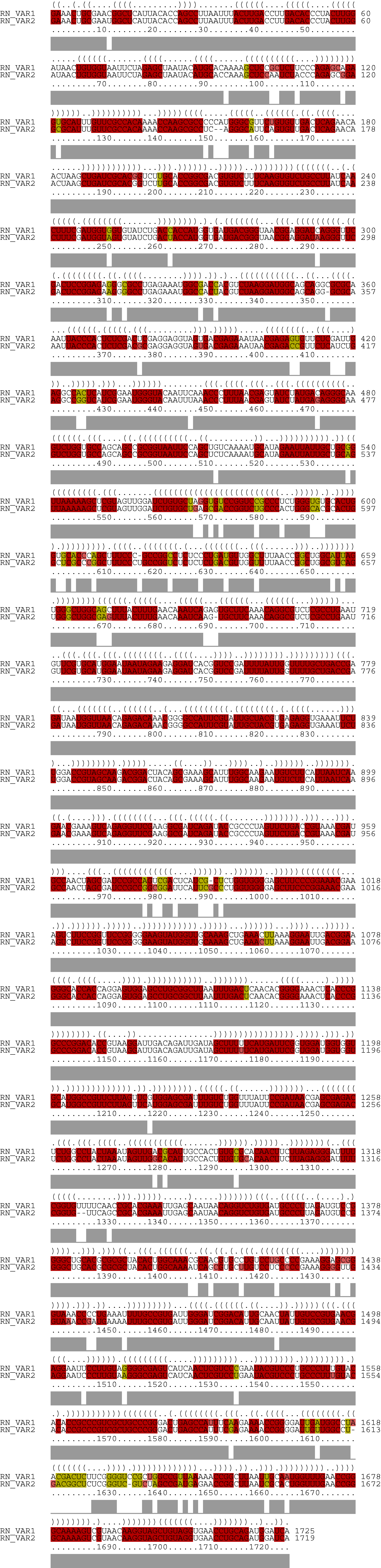

Supplement: Figure S3 — Multiple alignments of the 18S rDNA for RN_VAR1 and RN_VAR2. The bases highlighted in red indicate highly conserved areas between RN_VAR1 and RN_VAR2. The grooves in the grey bar represent dissimilarity among the nucleotides. (TIF) [file pone.0060891.s003.tif]

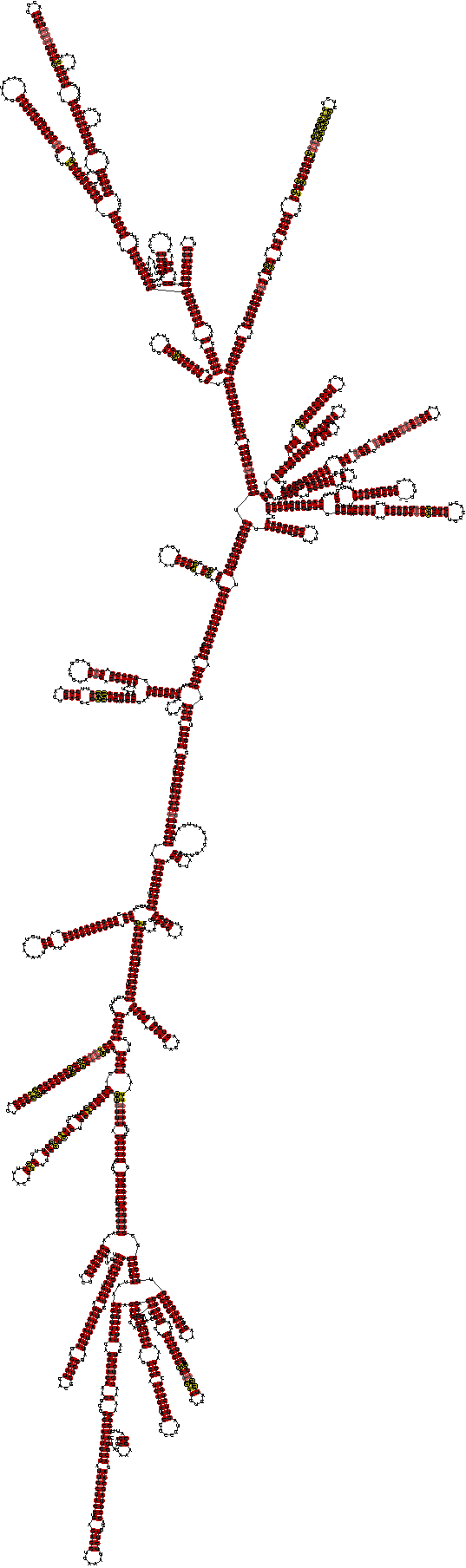

Supplement: Figure S4 — The consensus structure formed both by RN_VAR1 and RN_VAR2 together. (TIF) [file pone.0060891.s004.tif]
